# Multilocation Yield Trials and Yield Stability Evaluation by GGE Biplot Analysis of Promising Large-Seeded Peanut Lines

**DOI:** 10.3389/fgene.2022.876763

**Published:** 2022-08-04

**Authors:** Nipatcha Pobkhunthod, Jetsada Authapun, Songyos Chotchutima, Sarawut Rungmekarat, Piya Kittipadakul, Jaungjun Duangpatra, Tanapon Chaisan

**Affiliations:** ^1^ Agricultural Research and Technology Transfer Center, Faculty of Agriculture, Kasetsart University, Bangkok, Thailand; ^2^ Department of Agronomy, Faculty of Agriculture, Kasetsart University, Bangkok, Thailand

**Keywords:** yield stability, GGE biplot, GEI, pod yield, seed yield

## Abstract

The demand by industries for large-seeded peanuts is increasing in Thailand and Southeast Asia. New large-seeded peanut lines were recently developed in Thailand to respond to the demand. In this study, a multilocation yield trial was performed to identify the best genotype(s) in Thailand’s central region and investigate the genotype–environment interaction (GEI) on peanut production. Twelve promising large-seeded peanut lines and two check varieties (KU50 and KK6) were planted at 12 different planting locations during the dry and rainy seasons of 2018 and the dry season of 2019. This study found significant yield potential variability in the promising lines of peanuts evaluated at different planting locations. A combined analysis of variance presented that the environment and genotypes had a considerable impact (*p* < 0.001) on the pod and seed yield. The GEI showed a high impact (*p* < 0.01) on pod yield and an effect (*p* < 0.05) on seed yield. The environment presented the most significant influence on pod and seed yield variations, followed by genetics and GEI. The total variation in seed yield was 64.22%, composed of PC1 and PC2 values at 45.71% and 18.51%, respectively. The GGE biplot analysis of the yield potentials at each location indicated that KUP12BS029-1-1-3 was the ideal genotype, with a high yield potential and most stability at multilocations, followed by KUP12BS030-3-4-1 and KUP12BS030-1-4-3. These promising lines will be released as new peanut varieties in central Thailand and are recommended as parental lines in breeding programs for large-seeded and yield potential in Thailand and Southeast Asia.

## Introduction

Peanut (*Arachis hypogaea L.*) is an important legume crop in Southeast Asia. The market demand for large-seeded peanuts has been increasing, and better quality large-seeded peanut varieties are needed for peanut products, particularly by farmers. Therefore, breeding for yield has been the primary strategy to improve peanut productivity in peanut-growing countries (Nigam et al., 1991).

The peanut seed size is essential for the processed industry and can be used to measure the quality and price of peanuts. The size of the peanut seed indicates the quality of the peanut product. Peanut products made of large seeds taste better than those made of small seeds (Haruthaithanasan, 2002). The standard for grading peanut seeds are the following—1) large-seeded: 100-seeds weight of over 60 g, 2) medium-seeded: 100-seeds weight between 35 and 60 g, and 3) small-seeded: 100-kernels weight of less than 35 g (Waranyawat, 1999).

Most large-seeded peanut varieties have a late harvesting date, which is unsuitable for Thailand’s cropping system. The new large-seeded peanut varieties with resistance to peanut bud necrosis virus and early maturity date and suitable for the central region of Thailand were developed in 2010 under a peanut breeding program conducted by the Department of Agronomy, the Faculty of Agriculture, Kasetsart University, Thailand. Several large-seeded peanut genotypes were selected and evaluated at the research station in this peanut breeding program. A yield trial for the selected large-seeded peanut genotypes is required to determine their yield potential and stability at various planting locations (Authapun et al., 2016). This process is conducted in different environments over many years to measure the adaptability of the plants, identifying those that are potentially adaptable to specific environments and those that are generally adaptable (Yan and Hunt, 2001).

Selection for high yield and stability with critical economic traits, such as yield and adaptability, is essential in successful breeding programs. The necessary factors for consideration in breeding studies are variety, location, season, and environment (De lacy, 1981). Multi-environment or multilocation yield trials conducted in plant breeding programs are essential in evaluating genotypes and hybrids for yield and stability (Alwala et al., 2010). An essential factor in the stability studies of peanuts is the yield potential at multiple locations (Shorter and Norman, 1983). Generally, the yield of plants is influenced by the environment (E) more than the genotype–environment interaction (GEI) and genotype (G). Different peanut lines therefore show the highest yield potential at different locations (Tai and Hammons, 1978; Oliveira and Godoy, 2006; Kasno and Trustinah, 2015; De Moura et al., 2017). Testing plant varieties in different testing environments, where the responses would reflect the adaptability of the peanut genotypes to the inherent and persistent natural environmental factors of the different peanut production areas, provides valuable information for cultivar selection and final release (Banterng et al., 2006). Determining the most promising multilocation lines based on the findings of a preliminary yield trial, demonstrating yield potential and stability, is vital for selecting and releasing new varieties.

Different methods of analysis have been proposed to determine GEI, such as using a regression coefficient (Finlay and Wilkinson, 1963), calculating the sum of squared deviations from the regression (Eberhart and Russel, 1966), and the additive main effects and multiplicative interaction (AMMI) (Gauch and Zobel, 1988; Annicchiarico, 1997). Yan et al. (2000) proposed a method known as genotype plus genotype by environment interaction (GGE) or a multi-environment experiment in which the G + GE graph (GGE biplot) is displayed as a graph to facilitate the evaluation of the visible traits and the mega-environment identification of each genotype. The GGE biplot identifies the winning genotypes by crop every year. Therefore, using the GGE graph compares genotypes in different environments according to the best genotype and environmental performance, such as genotype grouping and environment. The polygon view of a GGE biplot explicitly displays the “which-won-where” pattern and hence provides a succinct summary of the GE pattern of a multi-environment trial data set. The polygon is formed by connecting the markers of the genotypes that are further away from the biplot origin such that all other genotypes are contained in the polygon (Yan, 2002).

The GGE biplot presents a graphic illustration of the data collected and aids in evaluating the comparative results. This biplot provided highly reliably graphical results to identify high yields and stability in genotypes in hybrid maize (Alwala et al., 2010). The biplot identified the mega-environment that influenced the variability of grain yield in barley (Jalata, 2011). It was used to select peanut germplasm for development as a specific genotype depending on environmental and management conditions (Zurweller et al., 2018). The objective of the current study was to apply a multilocation evaluation of large-seeded peanuts for yield stability. Specifically, the goals were to identify large-seeded peanut line(s) with high yield potential and stability suitable for multilocations.

## Materials and Methods

### Plant Materials

The peanut genotypes used in this study were 12 promising lines with a 100-seeds weight of more than 60 g. These lines were crossed from the Khon Kean 5 (KK5) variety (a Virginia-type peanut of medium seed size, high yield, a decumbent type canopy, and low incidence of bud necrosis virus disease) and Khon Kean 6 (KK6) variety (a Virginia-type, large-seeded peanut, with large pods, an erect plant type canopy, dark green leaves, and resistant to bud necrosis virus disease).

The two large-seeded check varieties were KK6 and Kasetsart 50 (KU50), recommended by the Thailand Department of Agriculture and often used by Thai farmers. KU50 is resistant to drought and foliar diseases and has a high yield and high seed dormancy.

### Multilocation Yield Trials

Twelve promising large-seeded peanut lines and two check varieties (KU50 and KK6) (Supplementary Table S1) were planted at twelve locations in central Thailand during the dry and rainy seasons of 2018 and the dry season of 2019 (Supplementary Table S2). The experiment at each location was conducted using a randomized complete block design with three replications. Crop management in this experiment included planting date, plant spacing, and irrigation at each location; each plot consisted of six rows and was 4 m long. Preemergent herbicides were used to control weeds in the experiments in all fields. All plots were fertilized during flowering at 156.25 kg·ha^−1^ of NPK (13-13-21). Gypsum (CaSO_4_·2H_2_O) was applied at early pegging at 312.50 kg·ha^−1^. The peanut fields were irrigated under drought conditions (Supplementary Table S2). The pod yield and seed yield were collected and reported at 8% moisture content.

### Weather and Soil Fertility

In the dry season of 2018, the average temperature ranged from 30.5°C to 30.9°C, and the total rainfall was approximately 166.3–380.6 mm. During the rainy season of 2018, the average temperature ranged from 27.7°C to 28.5°C, and the total rainfall was approximately 459.0–732.1 mm. During the dry season of 2019, the average temperature ranged from 30.7°C to 30.9°C, and the total rainfall was approximately 166.6–262.5 mm (Supplementary Table S2).

The planting area trials were conducted at 12 locations with known environmental differences, namely, 1) plains, 2) river plains, 3) piedmont plains, and 4) upland (Supplementary Table S2). The soil series in the planted areas were Nakhon Sawan, Lop Buri, Khok Samrong, Choke Chai, and Mae Sai. The Nakhon Sawan soil series is a shallow soil group to the rock wall layer. The soil reaction is acidic to neutral, with good drainage and low fertility. The Lop Buri soil series is an intense black clay soil group with deep and wide cracks when dry. The soil reaction in this soil series is neutral to alkaline, with moderate to good drainage and moderate to high fertility. The Khok Samrong soil series is an enthusiastic fine loam soil group. The soil reaction of this soil series is neutral to alkaline, drainage is quite inadequate to worse and fertility is moderate to low. The Choke Chai soil series is a deep to intense clay soil group produced from the fine mass parent material. The soil reaction of this soil series is strongly acidic and the drainage quite bad, with low fertility. The Maesai soil series is siltstone sandy soil that has risen from distributary sedimentation. The soil reaction of this soil series is neutral or basic, and drainage is quite bad and fertility is moderate to low.

### Data Collection

The pod and seed yields were determined based on sampling in the two center rows of each plot, excluding plants at the head and end of each row. The pod yield was based on sun-dried measurements to reduce the moisture content. Dry pod and seed weights were measured and recorded at 8% moisture content.

### Statistical Data Analysis

The collected data were analyzed using the R program version 3.6.1 [R Core Team (2019). R: A language and environment for statistical computing. R Foundation for Statistical Computing, Vienna, Austria. URL https://www.R-project.org/] for pod and seed yield variance at multiple locations. The analysis of variance was used to compare the means in each environment. Significant differences among the means of yields were compared using Fisher’s protected least significant difference (LSD) at the 5% level of probability. Evaluation for the yield potential and stability was performed based on a GGE biplot graphical user interface package (GGE biplot GUI) using principal component 1 (PC1) and PC2 scores. Visualization of “which-won-where/what” patterns for the multilocation yield trials was used to study genotypes (G) and genotype–environment interactions (GEI).

## Results

### Combined Analysis of Variance for Pod Yield and Seed Yield

The results showed that environment (E), genotype–environment interaction (GEI), and genotype (G) significantly affected pod and seed yields (Table 1). In particular, GEI highly affected the pod yield (*p* < 0.01) and had a significant effect on the seed yield (*p* < 0.05). The highest variations based on the percentage of total variability represented by the total sum of squares in pod and seed yields were 55.19% and 64.97%, respectively. The E factor had the most significant influence on pod and seed yield variations. The GEI variation had the second greatest significance with 15.07% (pod) and 10.17% (seed). The G variation was the least influential, with 4.31% (pod) and 2.81% (seed).

**TABLE 1:** Combined analysis of pod yield and seed yield of 14 peanut lines tested across 12 locations.

Source of variation	Df	Pod yield (t·ha^−1^)				Seed yield (t·ha^−1^)			
		SS	MS	%SS	Pr (>F)	SS	MS	%SS	Pr (>F)
Environment (E)	11	799.24	72.658***	55.19	<2.2e-16***	363.43	33.039***	64.97	<2.2e-16***
Rep: E	12	49.70	4.141***	3.43	6.096e-06***	19.75	1.645***	3.53	6.647e-08***
Genotype (G)	13	62.43	4.802***	4.31	1.558e-07***	15.75	1.212***	2.81	101e-05***
G×E	143	208.18	1.456**	14.38	0.004446**	56.94	0.398*	10.17	0.04893*
Pooled error	324	328.58	1.014	22.69		103.52	0.320	18.51	
Total	503	1448.13				559.39			

***, **, *: significant at 0.001, 0.01, and 0.05 probability levels, respectively.

Df, degrees of freedom; SS, sum of squares; MS, mean square.

### Correlation Coefficient Analysis

Correlations of yield components on the pod and seed yields were analyzed for the 12 planting locations. All characteristics significantly affected the yield. The number of seeds per plant and pods per plant showed a high correlation with the pod yield (*r* = 0.73, 0.70) and seed yield (*r* = 0.70, 0.53), respectively. However, the number of seeds per pod had a low correlation with the pod yield and seed yield (*r* = 0.04, 0.05). Similarly, 100-seeds weight had a low correlation with the pod yield (*r* = 0.03) (Table 2).

**TABLE 2:** Correlation coefficients of yield components with pod yield and seed yield of 14 peanut lines testing across 12 locations.

Yield component	Correlation coefficient	
	Pod yield	Seed yield
No. of pods per plant	0.70***	0.53***
No. of seeds per pod	0.04***	0.05***
No. of seeds per plant*	0.73***	0.70***
100-seeds weight (g)	0.19***	0.19***
Shelling percentage (%)	0.03***	0.15***

*; significant at 0.001 probability level.

### Yield Performance at Multilocations

The number of pods, seeds, 100-seeds weight, pod yield, and seed yield of the 14 large-seeded peanut lines in the 12 planting locations differed significantly (Table 3). The average pod yield differed significantly, ranging from 2.62 to 3.89 t·ha^−1^. The average pod yield of 14 large-seeded peanut lines at each planting location ranged from 0.90 [Wang Thong (WT)] to 4.95 t·ha^−1^ [Chon Phrai (CP)] (Table 4). The pod yield of the 14 large-seeded peanut lines was significantly different for 5 out of the 12 planting locations. The pod yield of KUP12BS029-1-1-3 (5.92 t·ha^−1^) was higher than that of KU50 (2.42 t·ha^−1^) at Lam Sonthi (LST). The pod yield of the KUP12BS030-4-2-1 (1.42 t·ha^−1^) peanut line was higher than that of KK6 (0.82 t·ha^−1^) and KU50 (0.53 t·ha^−1^) at WT.

**TABLE 3:** Yield and yield components of 12 peanut lines and 2 check varieties.

Code	Line/variety	No. pods per plant	No. seeds per plant	100-Seeds weight (g)	Pod yield (t·ha^−1^)	Relative to check		Relative to mean (%)	Seed yield (t·ha^−1^)	Relative to check		Relative to mean (%)
						KK6 (%)	KU50 (%)			KK6 (%)	KU50 (%)	
G1	KU50	30^bc^	47^bc^	56.14^d^	3.19^def^	92.78	100.00	95.15	1.41^b–e^	95.19	100.00	96.38
G2	KK6	27^cd^	40^cd^	64.81^bc^	3.41^a–e^	100.00	107.79	102.56	KK6	100.00^cd^	105.05^cd^	101.25^bc^
G3	KUP12BS001-3-4-3	31^b^	48^ab^	53.44^d^	3.00^efg^	87.35	94.15	89.59	1.62^cde^	90.19	94.75	91.32
G4	KUP12BS014-3-4-1	25^d^	41^cd^	55.37^d^	2.79^fg^	81.12	87.44	83.20	1.45^e^	80.54	84.61	81.55
G5	KUP12BS014-5-1-3	29^bc^	44^bcd^	52.71^d^	2.62^g^	76.32	82.26	78.27	1.50^de^	83.28	87.48	84.32
G6	KUP12BS029-1-1-3	35^a^	53^a^	63.64^bc^	3.89^a^	113.11	121.92	116.01	2.17^a^	120.70	126.80	122.21
G7	KUP12BS030-1-4-3	30^bc^	46^bc^	64.61^bc^	3.66^abc^	106.45	114.73	109.17	1.92^ab^	106.72	112.11	108.06
G8	KUP12BS030-3-4-1	30^bc^	43^bcd^	68.88^a^	3.52^a–d^	102.38	110.35	105.00	1.89^bc^	103.79	109.03	105.09
G9	KUP12BS030-4-2-1	30^bc^	45^bcd^	66.97^ab^	3.64^a–d^	105.82	114.06	108.53	1.95^ab^	108.59	114.07	109.94
G10	KUP12BS031-2-4-2	29^bc^	41^cd^	63.96^bc^	3.72^ab^	108.22	116.65	111.00	1.86^bc^	103.44	108.67	104.74
G11	KUP12BS031-5-2-1	26^cd^	39^d^	65.58^abc^	3.29^b-e^	95.59	103.03	98.04	1.73^bcd^	96.25	101.11	97.45
G12	KUP12BS036-4-2-3	30^bc^	39^d^	65.72^abc^	3.64^a-d^	105.77	114.00	108.48	1.75^b–e^	95.26	100.07	96.45
G13	KUP12BS050-2-4-2	29^bc^	42^bcd^	66.71^ab^	3.25^c–f^	94.49	101.84	96.91	1.78^bc^	99.37	104.39	100.61
G14	KUP12BS054-2-4-3	31^b^	45^bcd^	61.88^c^	3.29^b–e^	95.65	103.10	98.10	1.78^bc^	98.83	103.82	100.07
Mean		29.43	43.79	62.17	3.35	97.50	105.09	100.00	1.77	98.73	103.71	100.00
F-test		**	***	**	***				***			
CV (%)		31.04	34.74	15.57	32.84				34.31			
LSD (0.05)		4.48	5.66	4.07	0.46				0.26			

***, **: significant at 0.001 and 0.01 probability levels.

Means with the different lowercase superscripts (a–g) in the same column represent significant differences.

**TABLE 4:** Pod yield (t·ha^−1^) of 12 peanut lines and 2 check varieties planted in 12 locations.

Code	Line/variety	Location												
		CP	KC	KS	LST	PN	SB	ST	TF	TL	WM	WP	WT	Mean
G1	KU50	4.67	3.73^abc^	5.96^a–d^	2.42^c^	3.25	1.56	3.83	3.42	2.83	2.37	2.89^a–d^	0.53^d^	3.16
G2	KK6	6.04	5.38^ab^	7.05^ab^	3.88^abc^	2.05	1.58	3.77	3.62	1.35	2.82	3.40^ab^	0.82^bcd^	3.48
G3	KUP12BS001-3-4-3	5.65	4.38^abc^	5.20^-e^	2.43^c^	2.75	1.33	3.11	3.73	3.16	2.36	1.29^e^	0.69^cd^	3.01
G4	KUP12BS014-3-4-1	3.88	2.38^c^	3.30^a–e^	2.56^c^	2.53	2.49	2.64	3.56	2.81	2.97	2.00^cde^	0.42^d^	2.63
G5	KUP12BS014-5-1-3	4.87	3.13^bc^	3.99^de^	2.83^c^	2.62	1.27	3.82	2.77	2.71	2.87	1.78^cde^	0.87^bcd^	2.79
G6	KUP12BS029-1-1-3	5.26	4.71^abc^	6.18^abc^	5.92^a^	2.87	2.16	6.44	3.64	3.15	2.59	2.91^a–d^	0.89^bcd^	3.89
G7	KUP12BS030-1-4-3	5.18	3.67^bc^	6.11^a-d^	4.83^abc^	2.72	3.69	4.51	3.67	2.14	2.83	3.42^ab^	1.20^abc^	3.66
G8	KUP12BS030-3-4-1	4.79	5.05^ab^	5.22^a-e^	5.85^a^	2.26	1.89	4.21	3.06	3.30	2.46	2.96^a-d^	1.25^ab^	3.52
G9	KUP12BS030-4-2-1	4.95	4.58^abc^	4.23^de^	5.70^ab^	2.38	1.91	5.20	4.84	3.26	2.18	3.07^abc^	1.42^a^	3.64
G10	KUP12BS031-2-4-2	4.90	6.07^a^	7.06^ab^	3.46^c^	2.55	1.73	5.35	4.08	2.84	2.29	3.58^a^	0.80^bcd^	3.73
G11	KUP12BS031-5-2-1	5.41	4.56^abc^	4.84^b–e^	3.03^c^	2.36	2.18	4.27	3.64	2.97	2.15	3.40^b^	0.69^cd^	3.29
G12	KUP12BS036-4-2-3	5.06	5.11^ab^	7.46^a^	4.49^c^	2.79	2.29	4.49	3.49	2.64	2.85	2.09^cde^	0.94^a–d^	3.64
G13	KUP12BS050-2-4-2	4.29	4.62^abc^	4.84^b–e^	3.99^c^	2.50	1.93	3.97	4.35	2.88	2.40	2.47^a–e^	0.78^bcd^	3.25
G14	KUP12BS054-2-4-3	4.35	3.91^abc^	4.42^cde^	4.58^c^	2.44	2.53	4.72	3.77	2.73	2.53	2.25^b–e^	1.27^ab^	3.29
Mean		4.95	4.38	5.42	4.00	2.58	2.07	4.31	3.69	2.77	2.55	2.69	0.90	
F-test		ns	*	*	*	ns	ns	ns	ns	ns	ns	*	*	
CV (%)		9.34	32.60	25.01	37.14	18.83	43.79	32.86	24.75	31.54	16.3	26.75	34.27	
LSD (0.05)		1.39	2.35	2.25	2.45	0.71	1.59	2.33	1.58	1.57	0.69	1.59	0.50	

*: significant at 0.05 probability level; ns, not significant.

Means with the different lowercase superscripts (a–e) in the same column represent significant differences.

CP, Chon Phrai; KC, Khok Charoen; KS, Khok Samrong; LST, Lam Sonthi; PN, Phatthana Nikhom; SB, Sa Bot; ST, Si Thep; TF, Tak Fa; Tl, Tha Luang; WM, Wang Muang; WT, Wang Thong; WP, Wang Phloeng.

KUP12BS029-1-1-3 showed a high performance for the number of pods, number of seeds, pod yield, and seed yield. The average seed yields from the 12 planting locations were significantly different, ranging from 1.45 to 2.17 t·ha^−1^. The averaged seed yield of the KUP12BS029-1-1-3 line was higher than for that of KK6 (20.70%) and KU 50 (26.80%) (Table 3). At each planting location, the average seed yield ranged from 2.19 (WT) to 3.64 t·ha^−1^ (CP) (Table 5). The seed yield of the 14 large-seeded peanut lines in each planting area was significantly different in 3 out of the 12 planting locations at WP, Sa Bot (SB), and Wang Phloeng (WP). The seed yield of KUP12BS030-1-4-3 (1.26 t·ha^−1^) was higher than that for KU50 (0.82 t·ha^−1^) and KK6 (0.53 t·ha^−1^) at SB. The seed yield of KUP12BS030-4-2-1 (0.48 t·ha^−1^) was higher than that for KU50 (0.18 t·ha^−1^) and KK6 (0.25 t·ha^−1^) at WT. However, the seed yield of KUP12BS029-1-1-3 was not different from that of KK6 and KU 50 at each location.

**TABLE 5:** Seed yield (t·ha^−1^) of 12 peanut lines and 2 check varieties planted in 12 locations.

Code	Line/variety	Location												
		CP	KC	KS	LST	PN	SB	ST	TF	TL	WM	WP	WT	Mean
G1	KU50	4.06	1.59	3.57	2.32	1.15	0.53^b^	2.40	2.12	0.81	0.82	1.40^ab^	0.25^bcd^	1.75
G2	KK6	2.69	2.18	3.30	1.42	1.57	0.82^b^	2.46	2.05	1.66	1.56	1.25^abc^	0.18^cd^	1.76
G3	KUP12BS001-3-4-3	3.10	1.72	2.83	1.64	1.41	0.50^b^	1.92	2.03	2.05	1.49	0.57^d^	0.24^bcd^	1.62
G4	KUP12BS014-3-4-1	2.93	0.81	1.90	1.67	1.40	0.73^b^	1.70	2.16	1.78	1.41	0.92^bcd^	0.13^d^	1.46
G5	KUP12BS014-5-1-3	3.04	1.37	2.21	1.38	1.72	0.55^b^	2.49	1.60	1.44	1.23	0.67^d^	0.29^a–d^	1.50
G6	KUP12BS029-1-1-3	3.64	2.03	3.62	3.22	1.72	0.63^b^	4.36	2.30	1.92	1.15	1.22^abc^	0.29^a–d^	2.18
G7	KUP12BS030-1-4-3	3.13	1.46	3.18	2.68	1.65	1.26^a^	2.78	2.52	1.19	1.36	1.49^a^	0.38^abc^	1.92
G8	KUP12BS030-3-4-1	3.49	1.89	2.82	2.92	1.29	0.72^b^	2.76	1.75	1.91	1.26	1.31^abc^	0.33^a–d^	1.87
G9	KUP12BS030-4-2-1	3.05	1.74	2.23	3.17	1.52	0.54^b^	3.19	2.93	2.04	1.27	1.31^abc^	0.48^a^	1.96
G10	KUP12BS031-2-4-2	3.42	2.05	3.19	1.75	1.44	0.68^b^	3.14	2.42	1.52	1.11	1.40^ab^	0.24^bcd^	1.86
G11	KUP12BS031-5-2-1	3.74	1.65	2.10	1.61	1.09	0.70^b^	2.90	2.28	1.73	1.28	1.56^a^	0.17^cd^	1.73
G12	KUP12BS036-4-2-3	2.67	1.70	3.22	2.20	1.70	0.51^b^	2.66	2.01	1.55	1.32	0.80^cd^	0.24^bcd^	1.72
G13	KUP12BS050-2-4-2	3.02	1.76	2.88	2.49	1.53	0.72^b^	2.39	2.68	1.52	1.19	1.07^a–d^	0.22^bcd^	1.79
G14	KUP12BS054-2-4-3	2.99	1.64	2.40	2.54	1.40	0.84^b^	3.06	2.27	1.57	1.29	0.95^bcd^	0.42^ab^	1.78
Mean		3.21	1.69	2.82	2.21	1.47	0.70	2.73	2.22	1.62	.1.27	1.14	0.28	
F-test		ns	ns	ns	ns	ns	*	ns	ns	ns	ns	**	*	
CV (%)		14.85	34.47	29.63	38.27	18.83	27.36	35.28	20.50	32.71	28.10	26.99	46.42	
LSD (0.05)		0.80	0.95	1.49	1.46	0.54	0.36	1.53	0.77	0.93	0.58	0.36	0.21	

**, *: significant at 0.01 and 0.05 probability levels; ns, not significant.

Means with the different lowercase superscripts (a–d) in the same column represent significant differences.

CP, Chon Phrai; KC, Khok Charoen; KS, Khok Samrong; LST, Lam Sonthi; PN, Phatthana Nikhom; SB, Sa Bot; ST, Si Thep; TF, Tak Fa; Tl, Tha Luang; WM, Wang Muang; WT, Wang Thong; WP, Wang Phloeng.

### Genotype Plus Genotype by Environment Interaction Biplot Analysis

**FIGURE 1:**
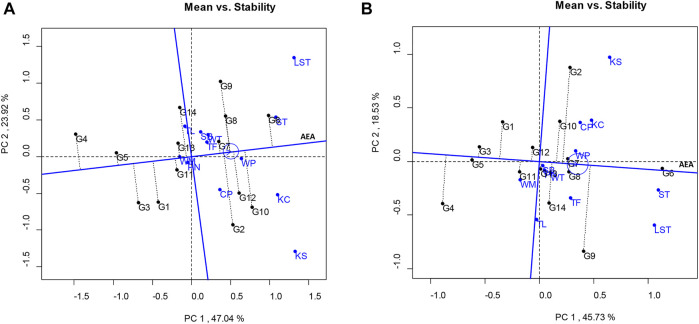
Average environment coordination views of the GGE biplot based on location-focused scaling of mean performance and stability of genotypes. **(A)** Pod yield. **(B)** Seed yield.

The results of the GGE biplot showed that the total variation of the pod yields was 70.96%, composed of PC1 (47.04%) and PC2 (23.92%) (Figure 1A). The PC1 score indicates the yield of the lines: PC1 > 0 indicates the high yield lines, whereas PC1 < 0 indicates the low yield lines. The PC2 score derived from the multilocation tests indicates a line’s stability. If the PC2 score approaches zero, the lines are stable. Based on the GGE biplot analysis, the peanut lines showed PC1 > 0 and low PC2 scores were KUP12BS030-1-4-3 (G7) and KUP12BS029-1-1-3 (G6), indicating high yield and high stability.

**FIGURE 2:**
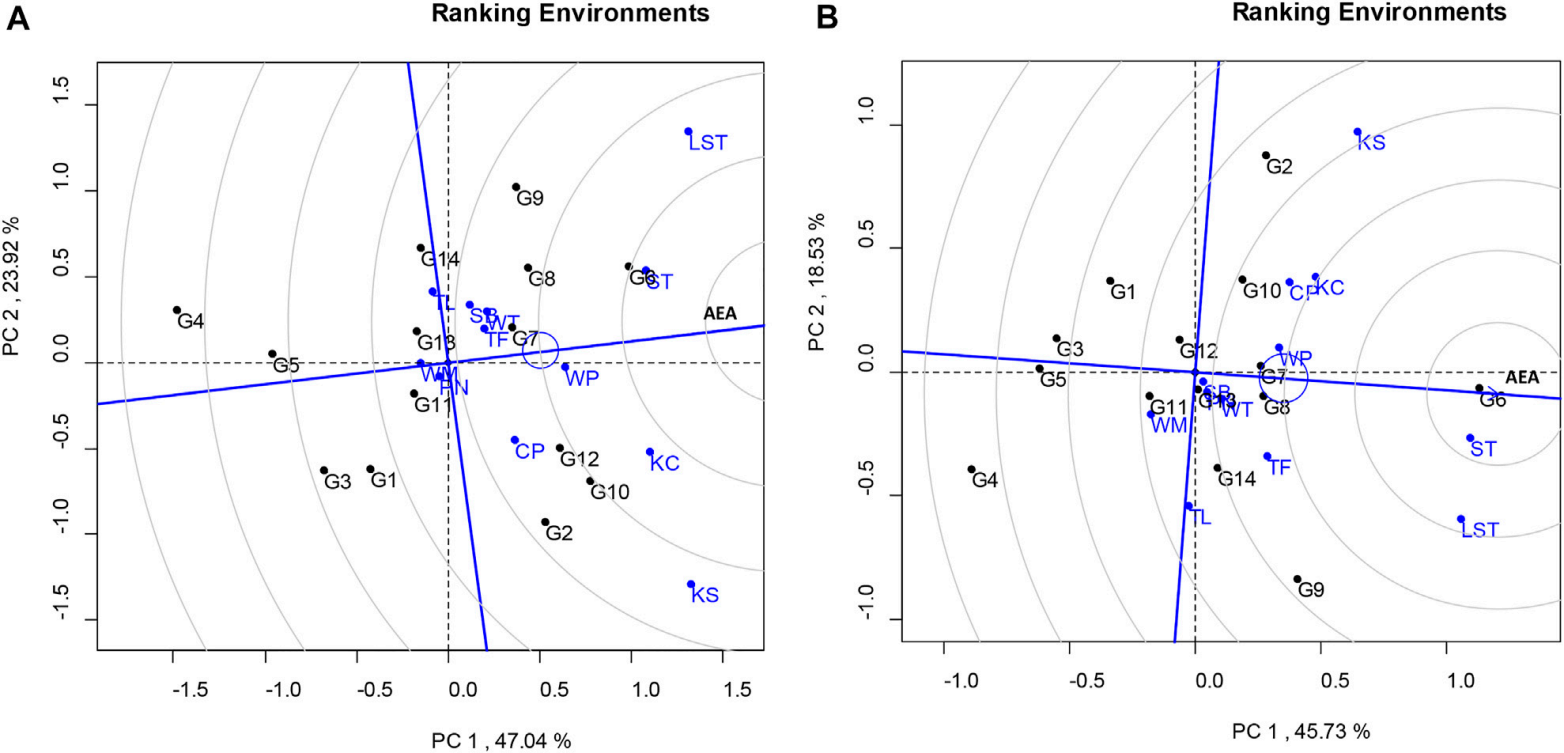
GGE biplot based on genotype-focused scaling comparing tested genotypes with ideal genotypes. **(A)** Pod yield. **(B)** Seed yield.

The total variation in the seed yield was 64.26%, composed of PC1 and PC2 values of 45.73% and 18.53%, respectively (Figure 1B). The lines that showed high stability and stable seed yield (PC1 > 0 and low PC2 score) were KUP12BS029-1-1-3 (G6), KUP12BS030-1-4-3 (G7), and KUP12BS030-3-4-1 (G8). KUP12BS029-1-1-3 (G6) had the highest mean pod yield and seed yield. Identification of the ideal genotype for pod and seed yields showed that KUP12BS029-1-1-3 (G6) was positioned the closest to the ideal pod and seed yield lines (Figure 2). Then, the KUP12BS029-1-1-3 (G6) was indicated as the ideal large-seeded peanut line for high stability and high pod and seed yields.

**FIGURE 3:**
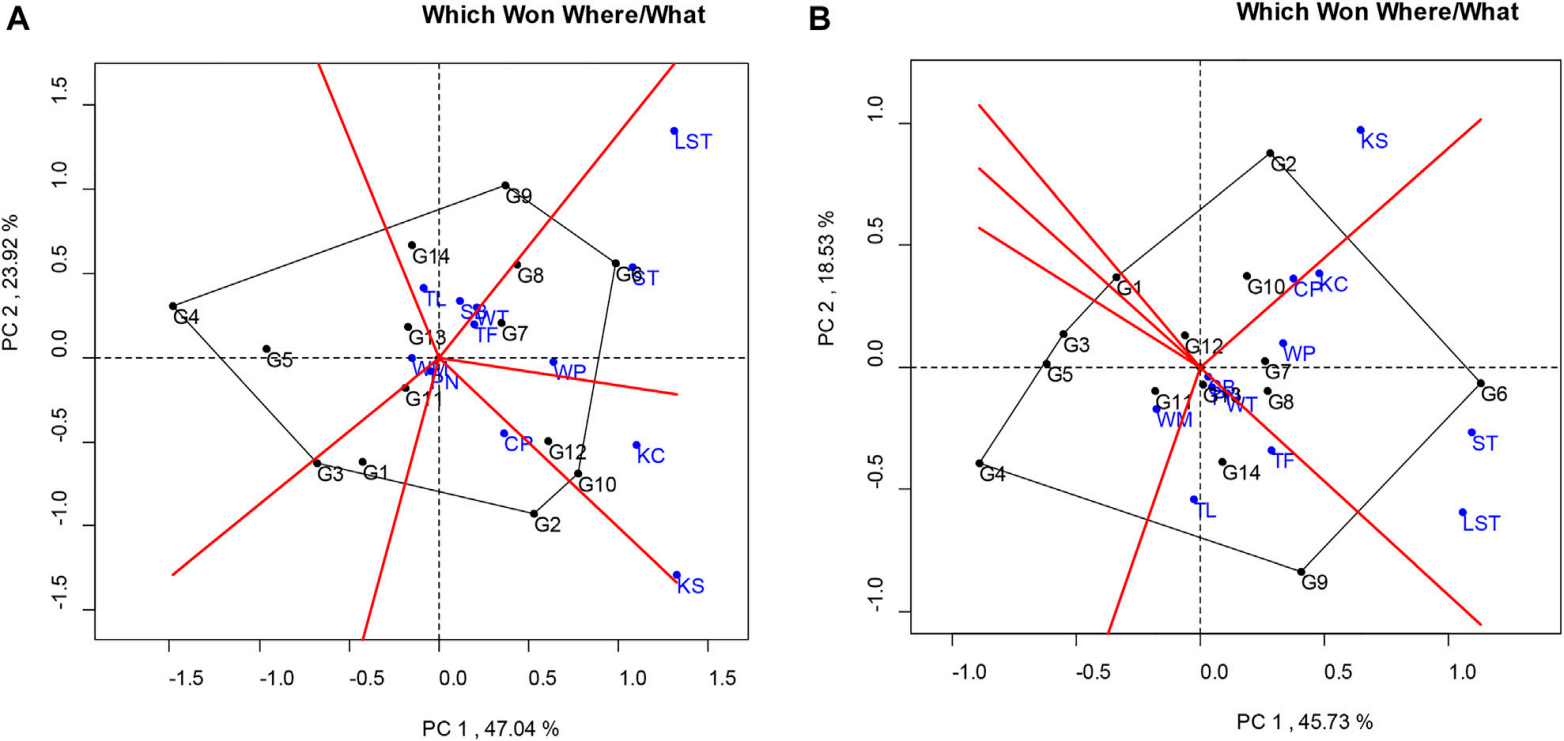
Polygon views of the GGE biplot of the “which-won-where/what” pattern of genotypes and locations. **(A)** Pod yield. **(B)** Seed yield.

The analysis for the pod yield of the peanut lines studied at multilocations was facilitated using a “which-won-where” pattern, showing the interaction of genotype to the data sets of different environments at the multilocation yield trials (Yan, 2002). The polygon view of this biplot showed the test locations in six sectors, where the lines at the corner of each section had the highest yield. The polygon view showed that the KUP12BS029-1-1-3 (G6) was the highest pod yield in Tak Fa (TF), Wang Phloeng (WP), Si Thep (ST), and Lam Sonthi (LST). KUP12BS030-4-2-1 (G9), KUP12BS001-3-4-3 (G3), KUP12BS014-3-4-1 (G4), and KK6 (G2) had the highest pod yields in Sa Bot (SB) and Tha Luang (TL), Phatthana Nikhom (PN), Wang Muang (WM), and Chon Phrai (CP), respectively (Figure 3A).

**FIGURE 4:**
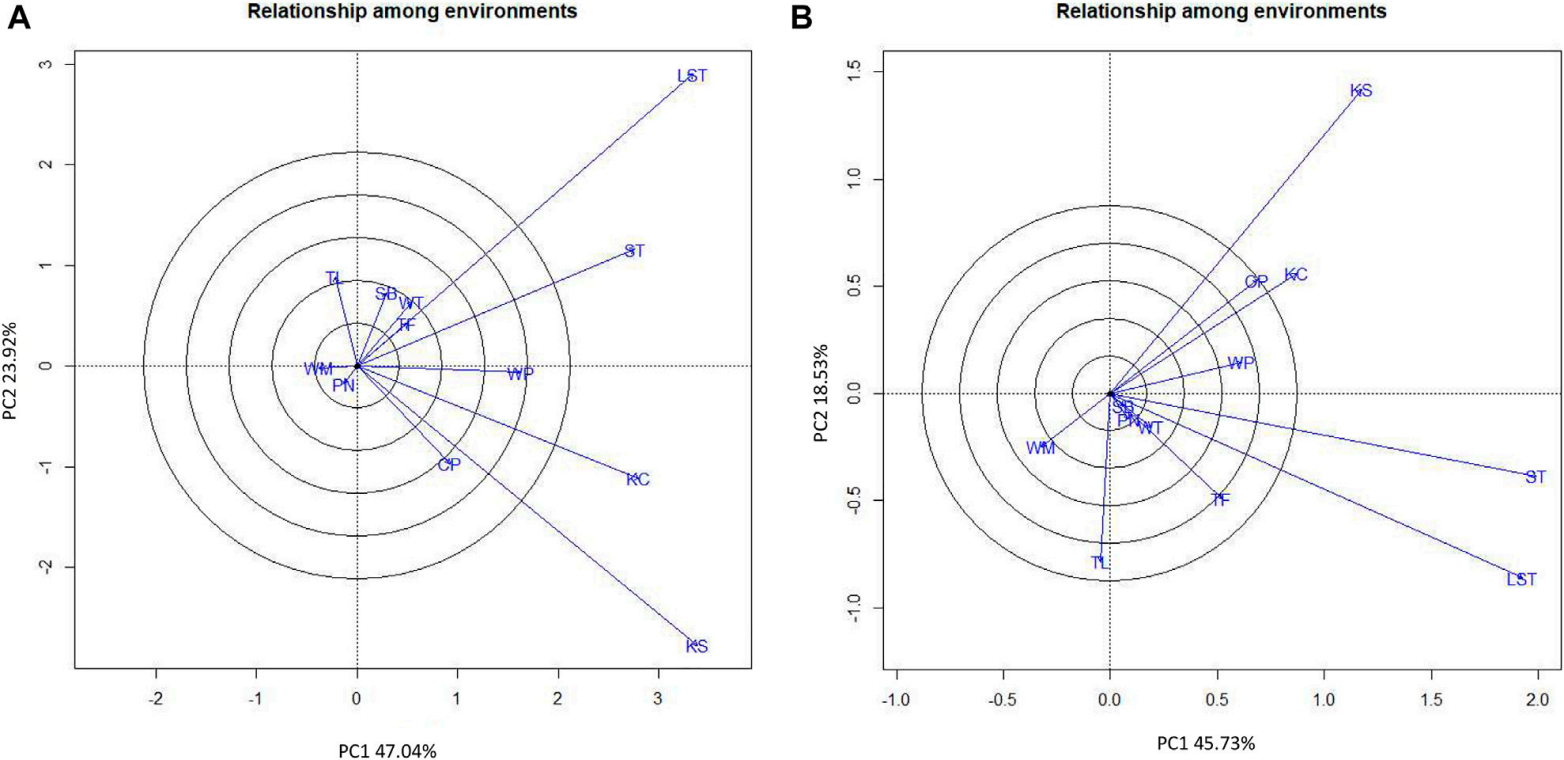
GGE biplot showing discriminating ability and representativeness of test locations. **(A)** Pod yield. **(B)** Seed yield.

Considering the seed yield of the lines at each location, the KUP12BS029-1-1-3 (G6) had the highest seed yield at Lam Sonthi (LST), Si Thep (ST), Wang Phloeng (WP), and Khok Charoen (KC) locations (Figure 3B). KUP12BS030-4-2-1 (G9) produced the highest seed yield in Tha Luang (TL), Tak Fa (TF), Wang Thong (WT), Phatthana Nikhom (PN), and Sa Bot (SB). KUP12BS014-3-4-1 (G4) showed the highest seed yield in Wang Muang (WM). Of the 12 testing locations, Lam Sonthi (LST) and Khok Samrong (KS), which had long vectors, were the most discriminating in pod yield, followed by Si Thep (ST) and Khok Charoen (KC) (Figure 4A), whereas Si Thep (ST) and Lam Sonthi (LST) were the most discriminating in seed yield (Figure 4B).

The average environment axis (AEA) of the biplot (Figure 4) is the line that passes through all locations represented by the circles and the center of the circles. The angle between the vectors at each location and AEA can be used to identify the representative location. A tested location with the smallest angle between its vector and AEA is the best representative of the test location (Yan and Tinker, 2006). Thus, ST (Si Thep) was the most representative location for both the pod yield and seed yield (Figure 4).

## Discussion

The combined analysis of variance showed significant effects for all sources of variation for both pod and seed yields, indicating the differential behavior of genotypes that were not consistent with the different environments. These results were consistent with other published reports of peanut yield trials (Dos Santo et al., 2012; Kasno and Trustinah, 2015). In the current study, the location influenced the large-seeded peanut lines. The pod yield and seed yield were the most critical factors affecting peanut yields. The influence of environmental variation on yield was more significant than GEI and genotype variations. Variations in the pod and seed yields of lines at the 12 locations were influenced mainly by the E effect, with minor influences by GEI and G. These results are consistent with reported GEIs in peanuts (Oliveira and Godoy, 2006; Kasno and Trustinah, 2015; De Moura et al., 2017). The variation of GEI is three times greater than the variation of G. Compared to G, the magnitude of GEI suggests that different environments might exist, which makes the breeder’s work with a selection more difficult. This result is consistent with earlier reports from a study of grain yield variation in barley caused by GEI (Jalata, 2011).

As the primary target for breeding in peanuts, the yield received more attention than the other examined traits. Correlation analyses revealed a pattern of association of traits and their direct contributions to yield. The correlation showed that the number of pods per plant and the number of seeds per plant had the most substantial positive direct effects on pod yield and seed yield, indicating that improvements in these traits should improve the total yield. These results follow reports regarding the number of pods per plant (Nandini and Savithramma, 2012; Thirumala et al., 2014). The 100-seeds weight and shelling percentage trait had a positive direct effect on yield, with weaker effects observed for the number of pods per plant and the number of seeds per plant trait. This information suggests that special attention should be paid to these traits when selecting high-yielding genotypes of large-seeded peanuts. The 100-seeds weight, based on the test planting data at 12 locations, found that some lines had 100-seeds weight less than 60 g, indicating the additive genetic effects and an opportunity to improve this trait *via* selection. Similar results were found for the peanut pod weight (Patil et al., 2014).

The current study identified that the rankings of the genotypes, based on yield, were changed for specific locations. For example, the pod yield of KUP12BS029-1-1-3, KUP12BS030-4-2-1, and KUP12BS036-4-2-3 presented the highest pod yield at LST, WT, and KS, respectively. These results presented the influence of GEI on the pod yield. GEI reduced the usefulness of lines by reducing their yield performance (Pham and Kang, 1988). Moreover, the pod and seed yield evaluations under multilocations showed different peanut lines presenting the highest pod yield at different locations. These results indicate that the influence of GEI had a crossover effect on pod and seed yields of these peanut lines. These results are consistent with earlier published reports of peanut yield trials (Banterng et al., 2006; Abhinandan et al., 2018; Zurweller et al., 2018; Okori et al., 2019). The mean yields observed in Chon Phrai were higher than in other sites. This result is not surprising when we consider that the Maesai soil series in the north of Chon Phrai is naturally more advantageous for this promising population.

The GGE analysis was performed to examine the performance and stability of promising lines and standard checks. This analysis was designed to provide an insight into the effect of G and GEI on yield and identify genotypes that are particularly well suited for a particular environment. It has been valuable for characterizing the broadly suitable locations for growing a specific line or group of genotypes. These GGE results indicated that the peanut lines KUP12BS029-1-1-3, KUP12BS030-4-2-1, and KUP12BS031-2-4-2 had the highest pod yields, whereas KUP12BS030-1-4-3 was the most stable peanut line. These results revealed that the line with the highest pod yield was not always the most stable line at each location. Thus, some large-seeded peanut lines showed high stability, but not all highly stable genotypes had a high mean pod and seed yield. Stability is only significant to farmers when this trait is associated with high mean performance (Jompuk, 2016).

Reviewing the biplot graph, KUP12BS029-1-1-3 had the highest yield at LST, ST, WP, TF, and WT, but KUP12BS029-1-1-3 had the highest yield at Lam Sonthi (LST) and Si Thep (ST) (Table 4). These results indicate that considering the multilocation data analysis using the GGE biplot method was more reliable than considering the data collected at each location (Yan, 2002). Lam Sonthi (LST) had long vectors and was the most discriminating for pod and seed yields. The nonrepresentative location can be considered a suitable testing location for selecting specifically adapted genotypes. However, the other locations with short vectors were not suitable for identifying peanut lines with high pod or seed yield (Yan and Tinker, 2006). For regional yield trials, the GGE biplot is an excellent analytical data tool for identifying the best genotypes at each location and the most stable genotypes for production. Moreover, this study reveals that the genetic and environment interaction (G×E) is the major barrier for increasing the yield of peanuts. According to the high effect of G×E, it is not easy to breed a new variety for use at different locations. For increasing the yield of large-seed peanuts, the breeder should focus on breeding a specific peanut variety for large-seed and high yields in a specific area.

## Conclusion

This study shows the variability of yields in large-seeded peanut lines at multiple locations. Locational variations influenced yield more than variations of GEI and G. For the testing locations, Si Thep (ST) was both discriminating and a representative location that can be considered a suitable testing location for selecting genotypes. The KUP12BS029-1-1-3 line presented a high yield at multilocations. Therefore, the KUP12BS029-1-1-3 line is the large-seeded peanut genotype that is most suitable and should be introduced to farmers because its genotype shows high yield and high stability at multiple locations. These promising large-seeded peanut lines are released as new peanut varieties and used as parental lines in breeding programs for large-seeded yields in Thailand and Southeast Asia.

## Data Availability

The original contributions presented in the study are included in the article/Supplementary Material, and further inquiries can be directed to the corresponding author.
