# Supplementary material for: Multilocation Yield Trials and Yield Stability Evaluation by GGE Biplot Analysis of Promising Large-Seeded Peanut Lines
Source: Front Genet. 2022 Aug 4;13:876763. doi: 10.3389/fgene.2022.876763 (PMC9386125; doi:10.3389/fgene.2022.876763)
Supplement: Supplementary file 1 [file DataSheet1.docx]

**Supplementary table 1**. **List of experimental plant materials**

| **Code** | **Line/Variety** | **Status** |
| --- | --- | --- |
| G1 | KK6 | Standard check |
| G2 | KU50 | Standard check |
| G3 | KUP12BS001-3-4-3 | Promising line |
| G4 | KUP12BS014-3-4-1 | Promising line |
| G5 | KUP12BS014-5-1-3 | Promising line |
| G6 | KUP12BS029-1-1-3 | Promising line |
| G7 | KUP12BS030-1-4-3 | Promising line |
| G8 | KUP12BS030-3-4-1 | Promising line |
| G9 | KUP12BS030-4-2-1 | Promising line |
| G10 | KUP12BS031-2-4-2 | Promising line |
| G11 | KUP12BS031-5-2-1 | Promising line |
| G12 | KUP12BS036-4-2-3 | Promising line |
| G13 | KUP12BS050-2-4-2 | Promising line |
| G14 | KUP12BS054-2-4-3 | Promising line |

**Supplementary table 2**. **Locations and weather conditions at multi location yield trials for each planting area**

| Locations | Planting date | Geographical coordinates | Land type | Soil type/  Soil series | Mean temp. (°C) | Rain fall  (mm) | Irrigation  (L ha^-1^) |
| --- | --- | --- | --- | --- | --- | --- | --- |
| 1. Wang Thong (WT) | Feb 12, 2018 | N15^o^26’37.6332 E100^o^52’8.778 | Flat area | Sandy loam/Ns | 30.8 | 252.1 | 3,432,000 |
| 2. Khok Charoen (KC) | Feb 12, 2018 | N15^o^37’951915 E100^o^82’84482 | Flat along the waterfront | Sandy loam /Ns | 30.8 | 295.6 | 2,640,000 |
| 3. Sa Bot (SB) | Feb 19, 2018 | N15^o^19515 E100^o^7804 | Flat area | Clay/Lb | 30.9 | 166.3 | 3,219,938 |
| 4. Wang Phloeng(WP) | Feb 22, 2018 | N15^o^1’41.4408E100^o^51’40.3164 | Upland area | Sandy loam /Ksr | 30.5 | 380.6 | 528,000 |
| 5. Tak Fa (TF) | Jul 31, 2018 | N15^o^28’35.7 E100^o^20’15.1 | Flat area | Clay/Lb | 28.3 | 459.0 | 2,837,313 |
| 6. Wang Muang (WM) | Aug 6, 2018 | N14^o^86’25.7 E101^o^11’66. | Flat area | Clay/Lb | 28.5 | 732.1 | 1,584,000 |
| 7. Phatthana Nikhom (PN) | Aug 15, 2018 | N14^o^45’57.9 E100^o^56’46.6 | Upland area | Silty Clay/Ci | 28.5 | 665.5 | 2,570,569 |
| 8. Chon Phrai (CP) | Sep 15, 2018 | N16^o^37.9151 E101^o^12.3779 | Upland area | Silty loam/Ms | 27.7 | 620.4 | 2,904,000 |
| 9. Si Thep (ST) | Feb 15, 2019 | N15^o^21’34.7 E101^o^14’58.0 | Upland area | Clay/Lb | 30.7 | 166.6 | 4,127,000 |
| 10. Tha Luang (Tl) | Feb 28, 2019 | N15^o^2’27’1716E101^o^18’32.6664 | Flat area | Clay/Lb | 30.6 | 201.5 | 3,869,063 |
| 11. Khok Sumrong (KS) | Feb 28, 2019 | N15^o^8’45682 E100^o^40’55862 | Flat area | Clay/Lb | 30.9 | 195.9 | 4,224,000 |
| 12. Lam Son Thi (LST) | Mar 12, 2019 | N15^o^50’49.6 E101^o^3438.7 | Flat area at the root of hill | Silty clay/Ci | 30.8 | 262.5 | 3,869,063 |

Soil series: Ns =Nakhon Sawan, Lb=Lop Buri, KSr= Khok Samrong, Ci=Choke Chai, Ms=Maesai

**Supplementary table 3. Analysis of variance for the number of pods per plant of 12 peanut lines and 2 check varieties at 12 locations**

| **Source** | df | SS | MS | F | Pr (>F) |
| --- | --- | --- | --- | --- | --- |
| Loc | 11 | 61053 | 5550.3 | 60.6482 | <2.2e-16*** |
| Line | 13 | 3144 | 241.9 | 2.6427 | 0.001601** |
| Loc:Line | 143 | 19718 | 137.9 | 1.5067 | 0.001534** |
| Loc:Rep | 12 | 3373 | 281.1 | 3.0715 | 0.000402*** |
| Residuals | 318 | 29102 | 91.5 |  |  |
| Total |  |  |  |  |  |

**Supplementary table 4. Analysis of variance for the number of seeds per plant of 12 peanut lines and 2 check varieties at 12 locations**

| **Source** | df | SS | MS | F | Pr (>F) |
| --- | --- | --- | --- | --- | --- |
| Loc | 11 | 140628 | 12784.4 | 61.4374 | <2.2e-16*** |
| Line | 13 | 6136 | 625.9 | 3.0078 | 0.0003443** |
| Loc:Line | 143 | 39400 | 275.5 | 1.3241 | 0.0216423* |
| Loc:Rep | 12 | 10322 | 860.2 | 4.1337 | 5.013e-06*** |
| Residuals | 319 | 66380 | 208.1 |  |  |
| Total |  |  |  |  |  |

**Supplementary table 5**. **Analysis of variance for the 100-seed weight of 12 peanut lines and 2 check varieties at 12 locations**

| **Source** | df | SS | MS | F | Pr (>F) |
| --- | --- | --- | --- | --- | --- |
| Loc | 11 | 30689 | 2789.92 | 36.8771 | <2.26-16*** |
| Line | 13 | 13698.4 | 1053.72 | 13.9281 | < 2.2e-16 *** |
| Loc:Line | 143 | 15987.8 | 111.80 | 1.4778 | 0.002347** |
| Loc:Rep | 12 | 4149.7 | 345.81 | 4.5709 | 7.81e-07*** |
| Residuals | 323 | 24436.4 | 75.65 |  |  |
| Total |  |  |  |  |  |

**Supplementary table 6**. **Analysis of variance for pod yield of 12 peanut lines and 2 check varieties at 12 locations**

| **Source** | df | SS | MS | F | Pr (>F) |
| --- | --- | --- | --- | --- | --- |
| Loc | 11 | 799.24 | 72.658 | 71.6448 | <2.2e-16*** |
| Line | 13 | 62.43 | 4.802 | 4.7354 | <1.558e-07 *** |
| Loc:Line | 143 | 208.18 | 1.456 | 1.4355 | 0.004446** |
| Loc:Rep | 12 | 49.70 | 4.141 | 4.0835 | 6.096e-06*** |
| Residuals | 324 | 328.58 | 1.014 |  |  |
| Total |  |  |  |  |  |

**Supplementary table 7**. **Analysis of variance for seed yield of 12 peanut lines and 2 check varieties at 12 locations**

| **Source** | df | SS | MS | F | | Pr (>F) | |
| --- | --- | --- | --- | --- | --- | --- | --- |
| Loc | 11 | 363.43 | 33.039 | 103.4070 | | <2.2e-16*** | |
| Line | 13 | 15.75 | 1.212 | 3.7928 | < 1.101e-05*** | |  |
| Loc:Line | 143 | 56.94 | 0.398 | 1.2462 | | 0.05615 | |
| Loc:Rep | 12 | 19750118 | 1.645 | 5.1497 | | 6.647e-08*** | |
| Residuals | 324 | 103.52 | 0.320 |  | |  | |
| Total |  |  |  |  | |  | |

**Supplementary table 8**. **Analysis of variance for pod yield at Khok Charoen (KC)**

| **Source** | df | SS | MS | F | Pr (>F) |
| --- | --- | --- | --- | --- | --- |
| Blk | 2 | 11.83 | 2.713 | 1.334 | 0.0725 |
| Line | 13 | 35.27 | 5.910 | 2.906 | 0.0459* |
| Eror | 26 | 52.89 | 2.034 |  |  |
| Total | 41 | 99.99 |  |  |  |

**Supplementary table 9**. **Analysis of variance for pod yield at Wang Thong (WT)**

| **Source** | df | SS | MS | F | Pr (>F) |
| --- | --- | --- | --- | --- | --- |
| Blk | 2 | 0.059 | 0.0297 | 0.309 | 0.7366 |
| Line | 13 | 3.302 | 0.25397 | 2.684 | 0.0155* |
| Eror | 26 | 2.460 | 0.09461 |  |  |
| Total | 41 | 5.821 |  |  |  |

**Supplementary table 10**. **Analysis of variance for pod yield at Sa Bot (SB)**

| **Source** | df | SS | MS | F | Pr (>F) |
| --- | --- | --- | --- | --- | --- |
| Blk | 2 | 5.249 | 2.6246 | 3.200 | 0.0572 |
| Line | 13 | 14.135 | 1.0873 | 1.327 | 0.2607 |
| Eror | 26 | 21.323 | 0.8201 |  |  |
| Total | 41 | 40.707 |  |  |  |

**Supplementary table 11**. **Analysis of variance for pod yield at Wang Phloeng (WP)**

| **Source** | df | SS | MS | F | Pr (>F) |
| --- | --- | --- | --- | --- | --- |
| Blk | 2 | 1.583 | 0.7916 | 1.541 | 0.23302 |
| Line | 13 | 19.552 | 1.5040 | 2.929 | 0.00951** |
| Eror | 26 | 13.353 | 0.5136 |  |  |
| Total | 41 | 34.488 |  |  |  |

**Supplementary table 12**. **Analysis of variance for pod yield at Tak Fa (TF)**

| **Source** | df | SS | MS | F | Pr (>F) |
| --- | --- | --- | --- | --- | --- |
| Blk | 2 | 10.146 | 5.073 | 6.078 | 0.00678 ** |
| Line | 13 | 9.917 | 0.763 | 0.916 | 0.55035 |
| Eror | 26 | 21.663 | 0.833 |  |  |
| Total | 41 | 41.726 |  |  |  |

**Supplementary table 13**. **Analysis of variance for pod yield at Wang Muang (WM)**

| **Source** | df | SS | MS | F | Pr (>F) |
| --- | --- | --- | --- | --- | --- |
| Blk | 2 | 3.442 | 1.7208 | 10.175 | 0.000544 *** |
| Line | 13 | 2.970 | 0.2285 | 1.351 | 0.263765 |
| Eror | 26 | 4.397 | 0.1691 |  |  |
| Total | 41 | 10.809 |  |  |  |

**Supplementary table 14**. **Analysis of variance for pod yield at Phatthana Nikhom (PN)**

| **Source** | df | SS | MS | F | Pr (>F) |
| --- | --- | --- | --- | --- | --- |
| Blk | 2 | 1.135 | 0.5674 | 3.717 | 0.038* |
| Line | 13 | 3.408 | 0.2622 | 1.718 | 0.117 |
| Eror | 26 | 3.968 | 0.1526 |  |  |
| Total | 41 | 8.511 |  |  |  |

**Supplementary table 15**. **Analysis of variance for pod yield at Chon Phrai (CP)**

| **Source** | df | SS | MS | F | Pr (>F) |
| --- | --- | --- | --- | --- | --- |
| Blk | 2 | 0.096 | 0.0480 | 0.066 | 0.936 |
| Line | 13 | 12.296 | 0.9459 | 1.311 | 0.263 |
| Eror | 26 | 18.579 | 0.7215 |  |  |
| Total | 41 | 30.971 |  |  |  |

**Supplementary table 16**. **Analysis of variance for pod yield at Si Thep (ST)**

| **Source** | df | SS | MS | F | Pr (>F) |
| --- | --- | --- | --- | --- | --- |
| Blk | 2 | 0.02 | 0.0077 | 0.004 | 0.996 |
| Line | 13 | 35.28 | 2.7136 | 1.346 | 0.251 |
| Eror | 26 | 52.43 | 2.0165 |  |  |
| Total | 41 |  |  |  |  |

**Supplementary table 17**. **Analysis of variance for pod yield at Khok Sumrong (KS)**

| **Source** | df | SS | MS | F | Pr (>F) |
| --- | --- | --- | --- | --- | --- |
| Blk | 2 | 1.42 | 0.711 | 0.382 | 0.6864 |
| Line | 13 | 61.65 | 4.742 | 2.546 | 0.0206* |
| Eror | 26 | 58.43 | 1.863 |  |  |
| Total | 41 | 121.50 |  |  |  |

**Supplementary table 18**. **Analysis of variance for pod yield at Tha Luang (TL)**

| **Source** | df | SS | MS | F | Pr (>F) |
| --- | --- | --- | --- | --- | --- |
| Blk | 2 | 21.726 | 10.863 | 14.231 | 6.69e-05*** |
| Line | 13 | 9.973 | 0.767 | 1.005 | 0474 |
| Eror | 26 | 19.846 | 0.763 |  |  |
| Total | 41 | 51.545 |  |  |  |

**Supplementary table 19**. **Analysis of variance for pod yield at Lam Son Thi (LST)**

| **Source** | df | SS | MS | F | Pr (>F) |
| --- | --- | --- | --- | --- | --- |
| Blk | 2 | 4.64 | 2.319 | 1.05 | 0.3643 |
| Line | 13 | 62.87 | 4.836 | 2.19 | 0.0431* |
| Eror | 26 | 57.42 | 2.208 |  |  |
| Total | 41 | 124.93 |  |  |  |

**Supplementary table 20**. **Analysis of variance for seed yield at Khok Charoen (KC)**

| **Source** | df | SS | MS | F | Pr (>F) |
| --- | --- | --- | --- | --- | --- |
| Blk | 2 | 1.599 | 0.7997 | 2.363 | 0.114 |
| Line | 13 | 4.430 | 0.348 | 1.007 | 0.473 |
| Eror | 26 | 8.799 | 0.3384 |  |  |
| Total | 41 | 14.828 |  |  |  |

**Supplementary table 21**. **Analysis of variance for seed yield at Wang Thong (WT)**

| **Source** | df | SS | MS | F | Pr (>F) |
| --- | --- | --- | --- | --- | --- |
| Blk | 2 | 0.0042 | 0.002088 | 0.125 | 0.883 |
| Line | 13 | 0.3820 | 0.029385 | 1.755 | 0.0108* |
| Eror | 26 | 0.4354 | 0.016745 |  |  |
| Total | 41 | 0.8216 |  |  |  |

**Supplementary table 22**. **Analysis of variance for seed yield at Sa Bot (SB)**

| **Source** | df | SS | MS | F | Pr (>F) |
| --- | --- | --- | --- | --- | --- |
| Blk | 2 | 0.4811 | 0.24056 | 6.660 | 0.00462** |
| Line | 13 | 1.5243 | 0.11725 | 3.246 | 0.00513** |
| Eror | 26 | 0.9392 | 0.03612 |  |  |
| Total | 41 | 2.9446 |  |  |  |

**Supplementary table 23**. **Analysis of variance for seed yield at Wang Phloeng(WP)**

| **Source** | df | SS | MS | F | Pr (>F) |
| --- | --- | --- | --- | --- | --- |
| Blk | 2 | 0.595 | 0.29757 | 3.175 | 0.05836 |
| Line | 13 | 3.781 | 0.29084 | 3.103 | 0.00675** |
| Eror | 26 | 2.437 | 0.09371 |  |  |
| Total | 41 | 6.813 |  |  |  |

**Supplementary table 24**. **Analysis of variance for seed yield at Tak Fa (TF)**

| **Source** | df | SS | MS | F | Pr (>F) |
| --- | --- | --- | --- | --- | --- |
| Blk | 2 | 4.291 | 2.1453 | 10.311 | 0.000505*** |
| Line | 13 | 4.765 | 0.3665 | 1.762 | 0.106268 |
| Eror | 26 | 5.410 | 0.281 |  |  |
| Total | 41 | 14.466 |  |  |  |

**Supplementary table 25**. **Analysis of variance for seed yield at Wang Muang (WM)**

| **Source** | df | SS | MS | F | Pr (>F) |
| --- | --- | --- | --- | --- | --- |
| Blk | 2 | 1.420 | 0.7102 | 5.571 | 0.00969** |
| Line | 13 | 1.249 | 0.0961 | 0.754 | 0.69754 |
| Eror | 26 | 3.315 | 0.1275 |  |  |
| Total | 41 | 5.984 |  |  |  |

**Supplementary table 26**. **Analysis of variance for seed yield at Phatthana Nikhom (PN)**

| **Source** | df | SS | MS | F | Pr (>F) |
| --- | --- | --- | --- | --- | --- |
| Blk | 2 | 0.9841 | 0.4921 | 6.416 | 0.00544** |
| Line | 13 | 1.5470 | 0.1190 | 1.552 | 0.16480 |
| Eror | 26 | 1.9941 | 0.0767 |  |  |
| Total | 41 | 4.5252 |  |  |  |

**Supplementary table 27**. **Analysis of variance for seed yield at Chon Phrai (CP)**

| **Source** | df | SS | MS | F | Pr (>F) |
| --- | --- | --- | --- | --- | --- |
| Blk | 2 | 0.378 | 0.1891 | 0.830 | 0.4473 |
| Line | 13 | 6.262 | 0.4817 | 2.114 | 0.0506 |
| Eror | 26 | 5.925 | 0.2279 |  |  |
| Total | 41 | 12.565 |  |  |  |

**Supplementary table 28**. **Analysis of variance for seed yield at Si Thep (ST)**

| **Source** | df | SS | MS | F | Pr (>F) |
| --- | --- | --- | --- | --- | --- |
| Blk | 2 | 0.113 | 0.0565 | 0.061 | 0.941 |
| Line | 13 | 15.773 | 1.2133 | 1.313 | 0.268 |
| Eror | 26 | 24.035 | 0.9244 |  |  |
| Total | 41 | 39.921 |  |  |  |

**Supplementary table 29**. **Analysis of variance for seed yield at Khok Sumrong (KS)**

| **Source** | df | SS | MS | F | Pr (>F) |
| --- | --- | --- | --- | --- | --- |
| Blk | 2 | 2.686 | 1.3431 | 1.929 | 0.166 |
| Line | 13 | 12.408 | 0.9545 | 1.371 | 0.238 |
| Eror | 26 | 18.101 | 0.6962 |  |  |
| Total | 41 | 33.195 |  |  |  |

**Supplementary table 30**. **Analysis of variance for seed yield at Tha Luang (TL)**

| **Source** | df | SS | MS | F | Pr (>F) |
| --- | --- | --- | --- | --- | --- |
| Blk | 2 | 11.901 | 5.951 | 21.155 | 3.52e-06*** |
| Line | 13 | 4.432 | 0.341 | 1.212 | 0.325 |
| Eror | 26 | 7.313 | 0.281 |  |  |
| Total | 41 | 23.646 |  |  |  |

**Supplementary table 31**. **Analysis of variance for seed yield at Lam Son Thi (LST)**

| **Source** | df | SS | MS | F | Pr (>F) |
| --- | --- | --- | --- | --- | --- |
| Blk | 2 | 1.463 | 0.7315 | 1.020 | 0.375 |
| Line | 13 | 16.140 | 1.2415 | 1.731 | 0.113 |
| Eror | 26 | 18.643 | 0.7170 |  |  |
| Total | 41 | 36.246 |  |  |  |
